# Supplementary material for: Dietary Intake Mendelian Randomization: Assessment and Development of Methods for Instrument Selection and Robust Inference
Source: medRxiv. 2025 Jun 27:2025.06.26.25330002. Preprint. [Version 1] doi: 10.1101/2025.06.26.25330002 (PMC12262755; doi:10.1101/2025.06.26.25330002)
Supplement: 1 [file NIHPP2025.06.26.25330002V1-supplement-1.pdf]

medRxiv preprint doi: https://doi.org/10.1101/2025.06.26.25330002; this version posted June 27, 2025. The copyright holder for this preprint (which was not certified by peer review) is the author/funder, who has granted medRxiv a license to display the preprint in perpetuity.
It is made available under a CC-BY 4.0 International license .

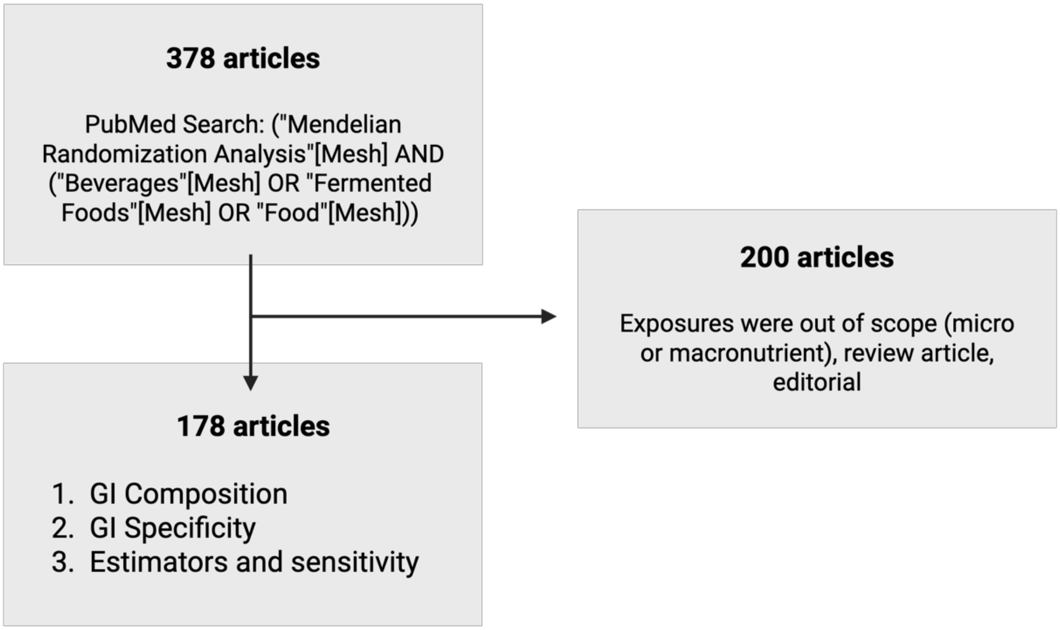

Supplemental Figure 1. Diagram of the studies identified and included from the assessment of the dietary intake MR literature up to January 24, 2025. Mesh, Medical Subject Headings; GI, genetic instrument.

medRxiv preprint doi: https://doi.org/10.1101/2025.06.26.25330002; this version posted June 27, 2025. The copyright holder for this preprint (which was not certified by peer review) is the author/funder, who has granted medRxiv a license to display the preprint in perpetuity.
It is made available under a CC-BY 4.0 International license .

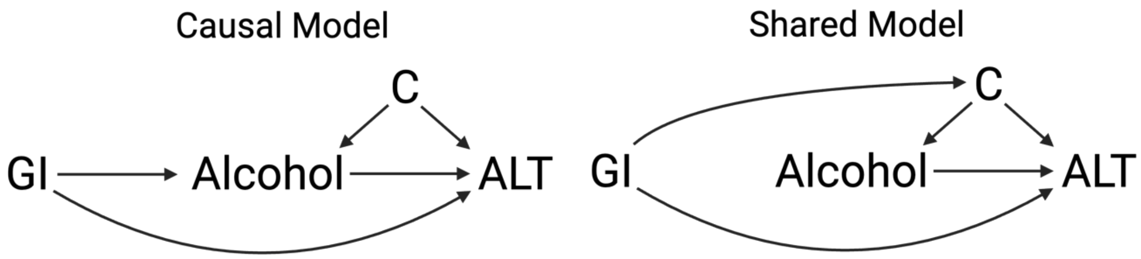

Supplemental Figure 2. Example models demonstrating the causal and shared models from MR-CAUSE. C, confounder.

medRxiv preprint doi: https://doi.org/10.1101/2025.06.26.25330002; this version posted June 27, 2025. The copyright holder for this preprint (which was not certified by peer review) is the author/funder, who has granted medRxiv a license to display the preprint in perpetuity.
It is made available under a CC-BY 4.0 International license .

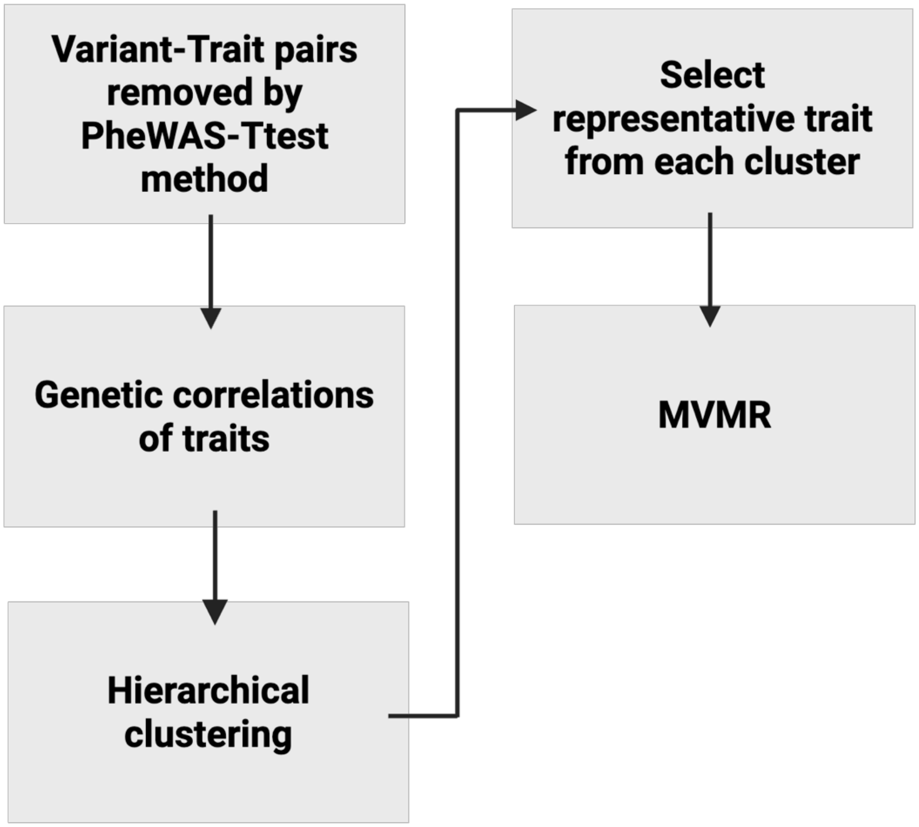

Supplemental Figure 3. Illustration of the pipeline that uses the output of the PheWAS-Ttest method to identify traits to include in MVMR mediation analysis to tease apart diet's direct and indirect paths on relevant health outcomes. The pipeline uses summary-level data, genetic correlation analysis, and hierarchical clustering to systematically select representative traits. MVMR, multivariable MR.
